# Dispersal and establishment of vascular epiphytes in human-modified landscapes

**DOI:** 10.1093/aobpla/plx052

**Published:** 2017-10-03

**Authors:** Helena J R Einzmann, Gerhard Zotz

**Affiliations:** 1 Department of Biology and Environmental Sciences, Carl von Ossietzky University of Oldenburg, Ammerländer Heerstraße 114–118, D-26129 Oldenburg, Germany; 2 Smithsonian Tropical Research Institute, Apartado Postal 0843-03092, Balboa, Ancon, Panamá, República de Panamá

**Keywords:** Fragmentation, land-use change, microclimate, seed dispersal, tropics

## Abstract

The ongoing destruction of old-growth forests puts tropical forest species under great pressure because of the resulting habitat loss. A pre-requisite for the maintenance of a viable metacommunity in a fragmented landscape is the connectivity between habitable patches. We experimentally studied four vital steps of epiphyte dispersal in different habitat types in western Panama. (i) Seed falling velocity (*V*_term_) is known to correlate with long-distance dispersal via convective updraft. All measured *V*_term_ of bromeliad and orchid seeds fell into a range of velocities with a high chance of long-distance dispersal. (ii) We quantified attachment success of bromeliad seeds as a function of bark rugosity with >30 common tree species in the region. Even fine bark structure allowed effective attachment. (iii and iv) Successful establishment is achieved when a seed germinates and a plantlet grows and survives. Germination success and early establishment of four bromeliad species did not differ between isolated trees, teak plantations or secondary forest patches. Microclimatic differences between habitat types were marginal and neither germination nor establishment correlated significantly with annual precipitation. The findings suggest a large capacity for dispersal and successful early establishment for these anemochorous species. A potentially regenerating forest may receive considerable input from sources such as pasture trees and in this way gain structural complexity, which also greatly enhances its value for other forest organisms.

## Teaser

The ongoing destruction of old-growth forests puts tropical forest species, with epiphytes as a key element, under great pressure. To maintain viable epiphyte communities in fragmented landscapes, remaining habitable patches have to be sufficiently connected. We experimentally studied four vital dispersal steps of vascular epiphytes in human modified-landscapes. Our findings suggest a high capacity for dispersal and successful early establishment for anemochorous species. Thus, potentially regenerating forests may receive considerable input from sources such as pasture trees and in this way gain structural complexity, which also greatly enhances their value for other forest organisms, such as canopy arthropods.

## Introduction

Increasing human populations lead to massive alteration and fragmentation of native vegetation that remains in patches within the landscape (cp. to Saunders *et al.* 1991). Although this is no new development at a local or regional scale, as example Forman (1987) quotes Plato (4th century B.C.) with a description of land overuse that left the land bare like a ‘mere skeleton’, but the current, global magnitude has led researchers to declare a global biodiversity crisis. In Central and South America, fragmentation of the remaining rainforests continues by conversion to agricultural land and wood removal (FAO 2011). This degradation of forest endangers all components of these ecosystems.

This contribution focuses on one of these biotic components, vascular epiphytes, which—by definition—depend on trees. Globally, they account for almost 10 % of all vascular plants (Zotz 2013), but locally, in humid montane forests they can represent up to 50 % of the vascular flora (Kelly *et al.* 2004). Epiphyte diversity is negatively affected by anthropogenic activities: the epiphytic habitats in modified landscapes, which typically consist of ‘islands’ of small natural forest fragments, secondary forests, tree plantations and scattered solitary trees, usually have reduced species richness and a modified floristic composition dominated by xerotolerant species (e.g. Barthlott *et al.* 2001; Krömer and Gradstein 2003; Köster *et al.* 2009; Larrea and Werner 2010). Together the assemblages inhabiting these ‘islands’ form a metacommunity. In such a fragmented environment seed dispersal is a particularly critical process (Bullock and Clarke 2000), because suitable safe sites are not necessarily close-by. In their review on the ‘ecology of seed dispersal’ Howe and Smallwood (1982) emphasize that the seeds of most species disperse only short distances on the order of tens of metres. In the particular case of epiphytes, seeds may be dispersed within a host tree, among neighbouring trees in a forest patch, between forest patches or into uninhabitable parts in between. For stochastic reasons local populations are typically bound to go extinct, but they can be rescued if they are connected with other populations which undergo asynchronous oscillations (Tremblay *et al.* 2006). To guarantee a constant renewal of epiphyte populations in a fragmented landscape, the habitat fragments have to be sufficiently connected. Thus, long-distance seed dispersal is of critical importance for epiphytes, and flora in general (e.g. Cain *et al.* 2000; Nathan *et al.* 2002; Bacles *et al.* 2006; Wright *et al.* 2008). In this study, we investigated the bottleneck of dispersal and establishment of vascular epiphytes in such a modified landscape by breaking down this early life stage into four consecutive steps.

Successful dispersal is a pre-requisite for long-term survival of epiphyte metacommunities. Since it is difficult to quantify long-distance seed dispersal directly this has been rarely done (but see Bullock and Clarke 2000). Dispersal of epiphytes has been assessed indirectly by monitoring the establishment of new individuals in the vicinity of a seed source (Werner and Gradstein 2008, 2009). Not surprisingly, seedling density was negatively correlated with distance to the source (distance to source was up to 300 m; Werner and Gradstein, 2008). In a study on the diversity of epiphytes along a human disturbance gradient Werner and Gradstein (2009) found no evidence for dispersal-limitation within a range of ca. 2 km from the seed source. An important advantage of epiphytes compared to ground-dwelling herbs is the elevated release point of the seeds, since height correlates with dispersal distance (Thomson *et al.* 2011). For very slowly falling seeds convective updrafts seem to be important for long-distance dispersal (Tackenberg *et al.* 2003). Convective updrafts occur especially in warm dry weather conditions and thus should be of importance for seed dispersal in the open landscape and warm tropical climate of the study area. As wind dispersal is typically facilitated in open habitat (Bacles *et al.* 2006) and since many of the large epiphytic taxa are entirely or largely anemochorous (e.g. orchids, bromeliads, ferns), they should actually benefit in this regard in open vegetation.

Dispersal will only be *effective* with subsequent establishment. For successful establishment the seeds need to attach to a substrate, where germination and seedling survival is possible (Winkler *et al.* 2005). Bark rugosity is assumed to facilitate attachment (Zotz 2016), hence bark type might represent an important factor for successful dispersal. Here, human action not only changes the spatial structure of hospitable habitats (host trees), but also the quality of hosts by introducing trees which are considered to be unsuited for epiphytes, for example eucalypts or pine (e.g. Boelter *et al.* 2011; Calviño-Cancela *et al.* 2013; Ardila *et al.* 2015). Tree plantations, per se, are potential refuges for epiphytes in landscapes otherwise dominated by agricultural land. Depending on the planted species, however, plantation trees could differ in the suitability depending on their bark rugosity. After attachment, the subsequent establishment of epiphytes may be affected by comparatively harsh microclimate conditions in human-modified landscapes (Didham and Lawton 1999; Gove *et al.* 2009; cp. to Hietz-Seifert *et al.* 1996). A recent study (Carvajal-Hernández *et al.* 2017) documented a reduction of fern species richness by 5–60 % and marked changes in species composition comparing fern diversity in natural forests and degraded and secondary forest. This coincided with an increase in mean annual temperature of only ca. 1 °C, but a reduction in relative air humidity by about 20 %. Small seedlings are challenged by desiccation, caused by high evaporation due to, for example increased radiation and increased forced convection. Thus, dryer climate in isolated trees and, to a lesser degree, in plantations might be particularly stressful during the early ontogenetic stage (e.g. Zotz et al. 2001; Werner and Gradstein 2008).

In this study, we addressed four important steps in the process of epiphyte dispersal and establishment in human-modified landscapes. (i) As seed falling velocity (*V*_term_) is known to correlate with long-distance dispersal via convective updraft, we assessed the potential for long-distance dispersal by determining the falling velocity of epiphytes. More specifically, we assessed whether the recently documented long-term changes in community structure in pasture trees (Einzmann and Zotz 2017) are related to dispersal, that is whether interspecific differences in colonization rates correlated inversely with *V*_term_. (ii) The role of bark rugosity on colonization success was studied by quantifying attachment success of epiphyte seeds as a function of bark structure for a range of tree species. We expected a positive correlation of attachment success and increasing bark rugosity. Finally, we tested (iii) germination and (iv) establishment success of four bromeliad species on isolated trees in pastures, in teak plantations and in trees of secondary forest patches that differ in annual precipitation. At each site, we also determined microenvironmental variables within tree crowns, expecting to find increased temperature and radiation but lower relative humidity in isolated trees compared to trees growing in plantations or secondary forest patches. The seeds used for these experiments were collected at sites with large differences in annual rainfall. For species from more humid sites we expected germination and establishment to differ at a local scale (germination on isolated trees < monoculture trees and secondary forest patches) and a regional scale (germination in dryer regions < more humid regions).

## Methods

Four sets of experiments were conducted which addressed different steps from dispersal to early establishment. (i) The determination of the falling velocity of seeds (*V*_term_) of various epiphyte species, (ii) an assessment of the ability of bromeliad seeds to get attached to bark, (iii) germination trials and (iv) an establishment experiment with four bromeliad species. The last two experiments were carried out in three different habitat types and at three levels of annual rainfall (Table 1). The assignment to one of the three rainfall levels was based on the data set of Poltz and Zotz (2011).

**Table 1.** Distribution and number of experimental sites of the germination and establishment experiments.

Habitat/host tree	Precipitation level (mm a^−1^)		
	≤1480	1520–3000	>3200
Isolated trees			
* Anacardium occidentale*	3	3	3
* Byrsonima crassifolia*	3	3	3
* Guazuma ulmifolia*	3	3	3
Plantation trees			
* Tectona grandis*	2	3	1
Undetermined forest species	3	2	2

Species names follow The Plant List (2013), and voucher specimens were deposited in the Herbarium of the University of Panama (PMA).

### Study area

The study was conducted on the Pacific slope of Panama in the lowlands of the Provinces Coclé, Herrera, Los Santos, Veraguas and Chiriquí. Agricultural land use has dominated for many decades and small local communities are scattered throughout the region. The current vegetation resembles tropical dry to wet savannah. Forest fragments are rare, usually small, and mostly of secondary nature. The region features a humidity gradient that spans from 1100 to 4200 mm annual precipitation with a dry season of approximately 3 months from January to March (Poltz and Zotz 2011). The mean annual temperature varies from 27 °C at the coast to 25 °C inland (Anonymous 1988). The driest part of Azuero peninsula is somewhat different with a 4–5 months dry season and a mean annual temperature of c. 28 °C (Heckadon-Moreno 2009).

### Seed dispersal success and falling velocity of seeds

If successful dispersal were the main process limiting colonization, one would expect a negative correlation of seed falling velocity (*V*_term_) and colonization success. With the data of two repeated epiphyte censuses 2005 and 2012/13 (Einzmann and Zotz 2017) we calculated a species-specific index of colonization success in which each newly colonized tree was counted as success in relation to the number of trees colonized by this species in the first census:

$$\frac{\text{Sum of newly colonized trees}\times \;100}{\text{Sum of colonized trees in}2005}$$ (1)

To determine *V*_term_ we collected seeds of 13 epiphyte species that were available at the time (Table 2). For five orchid species *V*_term_ was measured with a custom-built device that measures the passing of an object through two fan-shaped laser beams and calculates the velocity via the time difference (Zotz *et al.* 2016). Since orchid seeds are too small to measure their fall individually, a small quantity of seeds was put into the charging device. The individual seeds often clung together and fell in small groups. The largest group, being the heaviest, passes the lasers first. Determining the number of seeds in the largest group after each run allowed us to compare group size and measured *V*_term_. The relationship of seed group size and *V*_term_ follows a power function (Zotz *et al.* 2016). We estimated *V*_term_ for an individual seed of each species via a linear regression of the log transformed data **[see Supporting Information—Figure S1**]. For eight bromeliad species *V*_term_ was determined by letting seeds fall over a distance of 1.8 m and stopping the time manually. The test was conducted in a small laboratory to minimize air movement. Each individual seed was extracted from the capsule with a pincer and shaken in a standardized manner so the coma could unfold before releasing it.

**Table 2.** Colonization success and falling velocity of seeds of five orchid and eight bromeliad species. For calculation of colonization success see Methods section. For Orchidaceae *V*_term_ are derived from the regression equation of the respective power function solved for a single seed (**[see Supporting Information—Figure S1]**, *n* = number of data points). For Bromeliaceae *V*_term_ are means ± SD, *n* = sample size.

Taxon	Colonization success (%)	*V*_term_ (m s^−1^)	*n*
Orchidaceae			
* Brassavola nodosa* Lindl.	68	0.13	40
* Camaridium ochroleucum* Lindl.	188	0.22	30
* Dimerandra emarginata* (G.Mey.) Hoehne	52	0.17	20
* Encyclia stellata* (Lindl.) Schltr.	90	0.20	40
* Epidendrum difforme* Jacq.	48	0.08	40
Bromeliaceae			
* Catopsis nutans* (Sw.) Griseb.	288	0.16 ± 0.03	30
* Tillandsia balbisiana* Schult. & Schult.f.	29	0.21 ± 0.03	10
* Tillandsia caput-medusae* E.Morren	68	0.27 ± 0.05	10
* Tillandsia elongata* Kunth	45	0.22 ± 0.05	30
* Tillandsia fasciculata* Sw.	32	0.29 ± 0.07	20
* Tillandsia festucoides* Brongn. ex Mez	50	0.22 ± 0.06	10
* Tillandsia flexuosa* Sw.	53	0.25 ± 0.06	30
* Vriesea sanguinolenta* Cogn. & Marchal	35	0.31 ± 0.05	20

### Seed adherence to bark of different tree species

To test the ability of bromeliad seeds to cling to different bark types we used seeds of *Tillandsia elongata*, which where abundantly available at the time. We compared the ability of seeds of 10 bromeliad species to cling to bark of a single tree species [**see Supporting Information—Table S2**]. Given that we found no significant differences (Kruskal–Wallis test *P* = 0.28, df = 9, χ^2^ = 10.99), our results are probably also applicable to other anemochorous bromeliads. With a plastic bottle, some sponge fabric, a piece of tube and some string a container was built that could be attached to the tree trunks in order to create a windless space **[see Supporting Information—Figure S3**]. Only bark without moss or lichens was considered. A seed was put into the opening, a regular ball pump was then fixed to the tube and with one standardized push of the pump the seed was blown into the container and against the tree trunk. Thus, the focal bark area (57 cm^2^), the covered distance (10 cm) and the amount of air to carry the seed (125 mL) were standardized, although the curvature of the trunks produced some variation in the size of the target areas. We used 10 seeds on each individual tree and 5 trees per tree species. For each tree, we determined bark rugosity which we quantified as the mean of five systematic measurements of structure depth per trunk using a tape measure. In the case of very smooth bark without any macroscopic surface structure we set the value to 0.1 mm, that is a magnitude lower than the tape measure’s minimum accuracy. All tested trees were growing in villages and towns or on pastures. We tested mature individuals of 33 tree species that we encountered within the study region. Tree size, defined by diameter at breast height (dbh), ranged from 14–69.5 cm for *Crescentia cujete* to 70–206 cm for *Ficus* sp.

### Microclimate measurements

**Figure 1.**
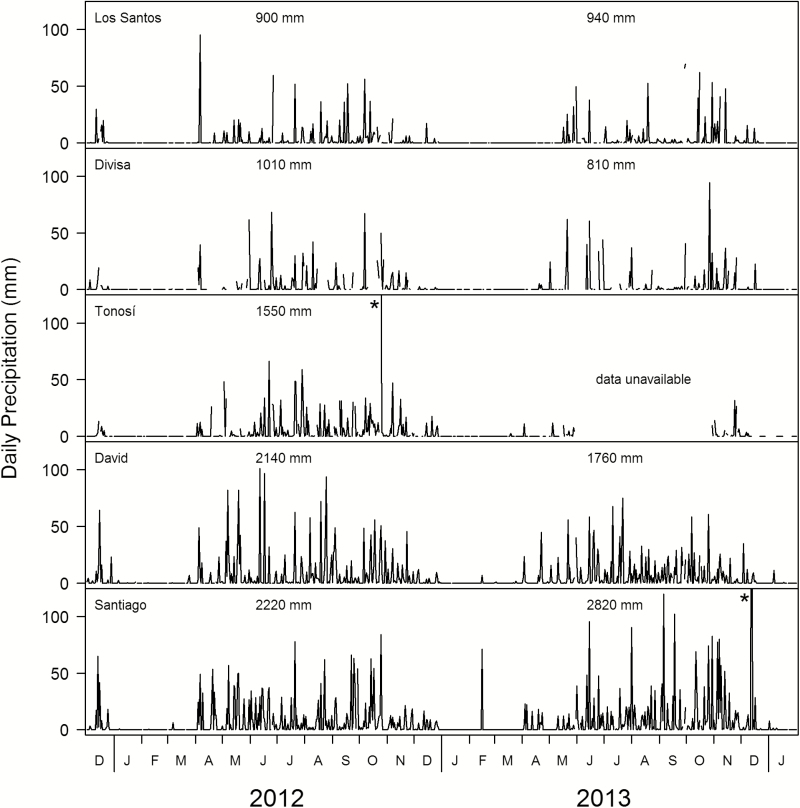
Daily precipitation during the study period for five cities within the study region. Extreme rainfall events (marked with an asterisk) in Santiago (360 mm on 12 December 2013) and Tonosí (almost 180 mm on 24 October 2012) are not fully displayed for better comparability of the plots. For Tonosí no data are available for almost the whole rainy season 2013. Figures within graph indicate annual precipitation of the respective years.

We measured microclimate variables from May 2012 to January 2014, representing one dry and two rainy seasons. The electricity supplier Empresa de Transmisión Eléctrica. S.A. (ETESA) provides climate data openly from their many gauging stations. Daily rainfall data are available for five cities within the study region. The differences in rainfall along the gradient become apparent in the varying length of dry seasons as well as overall amount of rainfall (Fig. 1). In all five places, dry season overlaps from January to the end of March, whereas at the dry end of the rainfall gradient the dry season already started in December and lasted into May. Therefore, we defined December, April and May as transition months that were excluded from the seasonal analyses of the obtained microclimate data. For our measurements, we used two devices that were mounted on a wooden structure that allowed a horizontal orientation of the light-measuring device and protected the relative humidity sensor from direct rain **[see Supporting Information—Figure S4A**]. The whole ensemble was fixed to the tree in the crown centre, corresponding roughly to Johansson Zone IV (Johansson 1974). Loggers were set up in the same trees of the three habitat types in which the germination and establishment experiments were conducted. However, in the isolated plots only one of the three species (*Anacardium occidentale*) could be included for longer periods because of the limited number of available loggers. In the other two species, microclimate was only studied episodically. Data and equipment loss caused further reductions in the number of cases to incorporate in the analysis, which led to rather uneven sample sizes (see number of days in Table 3).

**Table 3.** Microclimate within tree crowns of three different habitat types. Measurements were conducted in isolated trees, teak plantations, and secondary forest patches. Data from months of transition from dry to rainy seasons and vice versa were excluded. Data are means ± SD. Sample size is given as the number of days (No. days) separated for light and temperature and relative humidity (T-RH). For statistical treatment (see Supporting Information Fig. S7).

	No. days light	Light (klx)	No. days T-RH	Temp. (°C)	Relative humidity (%)	Minimum RH (%)
**Dry season**						
Isolated trees						
*Anacardium occidentale*	588	13.4 ± 6.2	688	26.8 ± 1.0	77.3 ± 8.5	54.2 ± 8.4
*Byrsonima crassifolia*	19	12.6 ± 3.3	21	26.6 ± 0.6	72.3 ± 9.1	53.9 ± 5.7
*Guazuma ulmifolia*	21	n.a.	40	26.3 ± 0.4	82.3 ± 2.9	56.1 ± 5.5
Plantation trees						
*Tectona grandis*	245	32.4 ± 8.8	259	27.0 ± 1.3	76.4 ± 8.6	47.6 ± 10.6
Undetermined forest trees	259	15.6 ± 6.4	307	26.1 ± 1.0	79.6 ± 6.5	59.3 ± 6.2
**Rainy season**						
Isolated trees						
*Anacardium occidentale*	2299	8.5 ± 5.5	2715	25.5 ± 0.9	92.7 ± 11.4	70.9 ± 19.8
*Byrsonima crassifolia*	183	4.3 ± 1.5	176	25.2 ± 0.7	97.0 ± 3.7	79.5 ± 7.4
*Guazuma ulmifolia*	359	9.5 ± 4.5	359	25.8 ± 0.8	94.2 ± 3.3	71.6 ± 7.4
Plantation trees						
*Tectona grandis*	1415	9.3 ± 5.6	1415	25.5 ± 0.9	91.3 ± 16.5	71.1 ± 24.4
Undetermined forest trees	1291	6.6 ± 3.4	1479	25.1 ± 1.1	95.6 ± 3.7	78.1 ± 8.6

The data loggers measured light (Hobo pendant, Onset Computer Corporation, Pocasset, MA), and temperature and relative humidity (Hobo U23 Pro V2, Onset Computer Corporation). The pendant loggers measure ambient light in lux within a broad wavelength spectrum (ca. 150–1200 nm). Thus, the data are not numerically comparable with photosynthetic active radiation, but in fact correlate closely with photon flux density (*r*^2^ = 0.88, Wagner *et al.* 2014). Loggers were calibrated against each other before and after the measuring campaign. Readings were taken every 24 min. The V2 loggers measured temperature and relative humidity every 30 min. Although the set-up shielded the V2 loggers from direct rain **[see Supporting Information—Figure S4A**], exposure to direct radiation occasionally occurred. This exposure can lead to recorded temperatures that differ substantially from ambient. Therefore, data points of temperature and relative humidity were removed manually when readings exceeded the mean by more than two times the standard deviation.

For calibration one logger was put next to the (calibrated) relative humidity sensor on the Lutz Tower microclimate monitoring station on BCI (Environmental Science Program (ESP); data download 2 June 2015) for 8 weeks. We regressed the data of our logger against the data of the ESP logger, and then used this logger as the reference for the calibration of all other V2 loggers. In rare cases, corrected relative humidity values slightly exceeded 100 %. For descriptive stats we kept these values. Statistical analyses were conducted with daily means of the microclimate variables.

### Germination and establishment of four bromeliad species in different habitat types along the rainfall gradient

For the establishment experiment seeds of *T. balbisiana*, *T. elongata*, *T. fasciculata* and *T. flexuosa* were collected from various sites within the study region in March 2012. These species were chosen due to their abundance in the study region and the need for a sufficient number of seeds for both the germination and the establishment experiment. The seeds were collected at two (*T. elongata* and *T. fasciculata*) or four sites (*T. balbisiana* and *T. flexuosa*), respectively. Infructescences yielded up to 55 capsules containing up to around 120 seeds. The capsules of each species were mixed and kept dry in paper bags until seeds were used. In 2013, fresh seeds were collected for the germination experiment. Both experiments were carried out in the three habitat types, that is secondary forest patches (*n* = 7), teak plantations (*n* = 6) and isolated trees (*n* = 9 × 3). In secondary forest patches and plantations, we haphazardly selected one tree for the experiments. For the isolated tree habitat, we selected three tree species that were abundant along the whole rainfall gradient of the study region: *Anacardium occidentale*, *Byrsonima crassifolia* and *Guazuma ulmifolia*. For each rainfall level (low: ≤1480 mm, medium: 1520–3000 mm, and high: >3200 mm annual precipitation) we set up the experiment at three sites. At these sites, individuals of the three tree species grew close to one another (mostly within a hectare and on one occasion within 1 km). As described above, we installed data loggers for light, temperature and relative humidity in *Anacardium* at each site.

All four epiphyte species are xerophytic members of the subfamily Tillandsioideae and use crassulacean acid metabolism (CAM) (Medina 1974, Griffiths and Smith 1983).Within the study region of the repeated census (Einzmann and Zotz 2017) the distribution of the species differed between the precipitation levels defined above. *Tillandsia balbisiana* and *T. fasciculata* did not occur in the plots that were in the area of low precipitation, but more than two-third of their individuals were found in plots with high precipitation. *Tillandsia elongata* was mainly found in plots with medium precipitation (85 % of its individuals), while almost half of the *T. flexuosa* individuals were found in plots with low precipitation, the other half was observed in plots with medium precipitation. We therefore expected germination and establishment on isolated trees and in areas of low humidity to be lowest in *T. balbisiana* and *T. fasciculata*.

#### Germination

In 2013, we set up four germination plots per tree oriented in the four cardinal directions. A plot represented an area of roughly 20 cm × 20 cm on a horizontal branch. If necessary, the plots were cleaned of coarse debris, mosses and lichens before the seeds were glued to the bark. Each plot contained 30 seeds per species. For easier handling, the seeds were tied together in bunches of 10 by their comas. Seed comas were glued to the bark with silicone drops (OBI Bau Silikon transparent, Aerotrim N.V., Overpelt, Belgium), assuring that the seeds themselves were free of silicone and touched the bark [**see Supporting Information—Figure S5A**]. Conspecific seeds were arranged in one line. The relative position of these lines was changed haphazardly in each plot. In January 2014, we revisited all the sites and counted the germinated plantlets that were still alive. In some cases, no trace of the seeds was found, either due to the loss of the whole package or due to possible predation (Zotz and Vollrath 2002, Chilpa-Galván *et al.* 2017). Germination success was calculated as percentage of the seeds still present in the tree. The loss of seeds was analysed separately.

#### Establishment

Seeds were collected in 2012 and were allowed to germinate in Petri dishes lined with cotton wool under constantly wet conditions, under shade cloth in the greenhouse facilities of the Smithsonian Tropical Research Institute (STRI) in Gamboa, Panama. After 3 months, plantlets were transferred to baskets of fine wire cloth, where they were allowed to grow for about 1 year. During their first month on the wire cloth they were irrigated weekly. Afterwards they received only water through natural rain events. Between 1500 (*T. fasciculata*) and 3000 plantlets (*T. flexuosa*) were available in 2013. The establishment experiment was conducted next to the plots of the germination experiment. To be able to distribute the plantlets more or less evenly between the plots we regrouped them. First, they were detached from the wire mesh. Then, we put 4–6 cm long silicon strands (OBI Bau Silikon transparent, Aerotrim N.V.) on the wire mesh in which we set 7–14 plantlets according to availability. These prepared units were then glued to the bark of the experimental trees, again with silicone [**see Supporting Information—Figure S5B**]. The relative position of the units was changed haphazardly in each plot. In January 2014, all sites were revisited and the surviving plantlets counted. Similar to the loss of seed packages the complete loss of seedlings was noted and analysed separately for survival.

### Data analysis

All statistical analyses were performed with R 3.0.2 (R Core Team 2013). If not stated otherwise, means are accompanied by ± standard deviation (SD). The falling velocity of seeds was related to colonization using a linear model. The effect of tree species on seed adherence to bark was tested with an ANOVA. Normal distribution of the percent data was achieved by angular transformation (Dytham 2003). Mean percent seed adherence was tested for correlation with bark rugosity applying the Kendall method. Moreover, for tree species with information on epiphyte abundance (Einzmann and Zotz 2017) we tested if seed adherence correlated with average relative abundance of epiphytes and the average gain of species and individuals from 2005 to 2013 as an indicator for new colonizations. The frequency distribution of seed adherence was strongly skewed. Therefore, we followed Chebyshev’s theorem that states that at most a quarter of all values lie beyond 2 SD of the mean and set the confidence intervals at 75 % (Kvanli *et al.* 2005). Lacking normal distribution, the microclimate data were analysed with a Kruskal–Wallis test (KW), followed by pairwise comparisons using Nemenyi-tests with chi-squared approximation for independent samples (NT, Pohlert 2014). The data were analysed separately for dry and rainy seasons. As the length of the seasons varied along the rainfall gradient we excluded the transition months between dry and rainy season from the analysis. Thus, for the dry season we only took data from January through March and for the rainy season we used data from June through November. Similar to the microclimate data, data of germination and establishment experiments were analysed using a KW test.

## Results

### Seed dispersal success and falling velocity of seeds

The relationship of *V*_term_ and colonization success for orchids and bromeliads did not meet expectations (Table 2). In the case of orchids, there was even a positive trend between colonization success and *V*_term_ (linear model: *F*_1,3_ = 3.5, *P* = 0.16, adjusted *r*^2^ = 0.4), whereas the negative correlation for bromeliads depended entirely on the inclusion of *Catopsis nutans* in the model. Since the extremely high colonization success of this species drove the whole model, we excluded *Catopsis* from this analysis, resulting in a non-significant relationship (linear model: *F*_1,5_ = 0.01, *P* = 0.9, adjusted *r*^2^ = −0.2).

### Seed adherence to bark of different tree species

Seed adherence was tested on 33 tree species belonging to 20 families, one tree species remained undetermined **[see Supporting Information—Table S6]**. Bark rugosity varied from almost zero (*Eucalyptus* sp and *Ficus* sp) to about 9 mm (*Pinus caribaea*).

**Figure 2.**
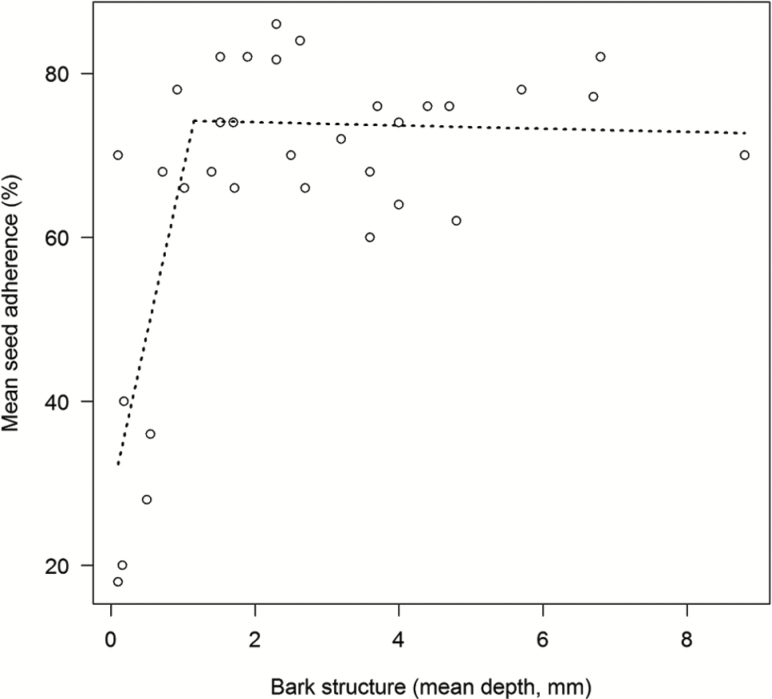
Segmented regression of mean seed adherence and bark rugosity of 33 tree species. Within the first segment there was a significant increase of mean seed adherence with rugosity. Data are means (*n* = 5). For error terms **[see Supporting Information—Table S6]**.

The relationship of seed adherence and bark rugosity was analysed with a segmented regression (Fig. 2). The model estimated the breakpoint at 1.2 ± 0.2 mm. In the first segment mean seed adherence increased significantly with rugosity (segmented linear model: *P* < 0.01, df = 29, adjusted *r*^2^ = 0.57). There was no correlation of seed adherence with relative abundance of epiphytes hosted by a tree species (Kendall’s rank correlation: *Z* = 1.0, *P* = 0.2, τ = 0.2), nor with average epiphyte species gain in the period from 2005 to 2013 (Kendall’s rank correlation: *Z* = 0.26, *P* = 0.4, τ = 0.04). Seed adherence did not differ with habitat type as seeds stuck on plantation tree bark (teak and pine) just as well as on 75 % of all tested native trees. Unsurprisingly, adherence was lowest in eucalypt with its particularly smooth bark (mean seed adherence 18 ± 17 %).

### Microclimate in tree crowns in three different habitat types

We measured microclimate variables within the tree crowns over more than 1.5 years. Differences in microclimate between the three habitat types were rather small even in the dry season, for example as light incidence in isolated trees and in forests differed only about 2 klx, temperature varied by just 1 °C and minimum relative humidity ranged between 50 % and 60 % for isolated trees and trees in secondary forests (Table 3). Also, isolated and plantation trees did not show consistent differences in terms of incident light, temperature and relative humidity compared to trees in secondary forest patches (Table 3 and **[see Supporting Information—Figure S7]**).

### Germination in different habitat types along the rainfall gradient

The germination experiment was conducted on trees in secondary forests, teak plantations and pastures (isolated trees). There were more than 3-fold differences (KW: χ^2^ = 23.2, df = 3, *P* < 0.001) with the lowest values in *T. fasciculata* (8 ± 9 %) compared to *T. elongata* (18 ± 15 %; NT: *P* < 0.05), *T. flexuosa* (29 ± 21 %; NT: *P* < 0.001), and *T. balbisiana* (16 ± 16 %, NT: *P* = 0.1). Habitat type did not significantly affect germination success in any of the four *Tillandsia* species (KW: *P* > 0.2), neither did tree species identity (KW: *P* > 0.3).

No difference in germination success was found when testing different rainfall levels among the forest plots for any of the species (KW: df = 2, *P* > 0.1), nor among teak plantations (KW: df = 2, *P* > 0.1), or pastures (KW: df = 2, *P* > 0.13—except *T. flexuosa* KW: χ^2^ = 5.8, df = 2, *P* = 0.06), although germination success in *T. flexuosa* tended to be low in high precipitation areas.

Loss of seeds was least in *Anacardium* (2 ± 4 %), which was significantly less compared to all others except *Byrsonima* (12 ± 18 %, KW: χ^2^ = 26.6, df = 4, *P* < 0.001; NT: *P* = 0.09): *Guazuma* (13 ± 16 %), forest trees (15 ± 14 %), and teak (21 ± 23 %, all NT: *P* < 0.05).

### Establishment in different habitat types along the rainfall gradient

Like germination, percent survival from the first to the second year of 1-year-old seedlings differed significantly between species (KW: χ^2^ = 37.3, df = 3, *P* < 0.001). Survival in *T. fasciculata* (27 ± 24 %) was significantly lower than in *T. balbisiana* (62 ± 27 %; NT: *P* < 0.001) and *T. elongata* (63 ± 24 %; NT: *P* < 0.001) but indistinguishable from that of *T. flexuosa* (41 ± 34 %; NT: *P* = 0.2). The only other significant difference in survival was found between *T. flexuosa* and *T. elongata* (NT: *P* < 0.05). Again, the three habitat types did not affect survival in any of the four species (KW: *P* > 0.19), although *T. fasciculata* showed a trend to higher survival in isolated trees and plantations. There was also no significant difference in survival of the species in the different tree species tested (KW: *P* > 0.4).

Survival did not vary with rainfall level in any species, neither in secondary forest plots (KW: df = 2, *P* > 0.1), nor teak plantations (KW: df = 1, *P* > 0.07). In pasture plots, survival of *T. fasciculata* and *T. flexuosa* was higher in the driest areas compared to the most humid ones (KW: χ^2^ = 12.9, df = 2, *P* < 0.01; NT: *P* < 0.05; KW: χ^2^ = 10.8, df = 2, *P* < 0.01; NT: *P* < 0.05). Differences in the other two species were not significant (KW: df = 2, *P* > 0.1).

Loss of entire plant packages did not differ between tree species (KW: χ^2^ = 10.0, df = 4, *P* < 0.05; NT: *P* > 0.05; all NT > 0.1).

## Discussion

Finding epiphytes in diverse human-modified habitats, from secondary forests to plantations to single trees in urban settings (Haro-Carrión *et al.* 2009; Köster *et al.* 2011; Poltz and Zotz 2011; Adhikari *et al.* 2012; Izuddin and Webb 2015; Einzmann *et al.* 2016) does not necessarily guarantee long-term persistence. Are populations sufficiently connected to rescue failing local populations in a metacommunity framework? Among the possible refuges in human-modified landscapes there is an increasing number of plantations with allochthonous trees which begs the question of their role as potential stepping stones. With this study, we focussed on the early live stages of epiphytes and their potential to disperse and establish successfully in three different habitats in human-modified landscape.

As most vascular epiphyte species are orchids (68 %, Zotz 2013) and virtually all orchids are wind-dispersed it is obvious that wind is the most important dispersal agent. Anemochorous plant species may benefit from open vegetation (Jesus *et al.* 2012) and some bromeliads are indeed more abundant in disturbed sites (Barthlott *et al.* 2001; Flores-Palacios and García-Franco 2004). Connectivity in fragmented landscapes does not appear to affect the probability of colonization in the case of epiphytic *bryophytes* either, which are also wind-dispersed (Snäll *et al.* 2003). Epiphyte seed dispersal benefits from their elevated growing sites in trees (Thomson *et al.* 2011). A slow seed falling velocity (<0.5–1.0 m s^−1^, Tackenberg *et al.* 2003) has been linked to higher chances of long-distance dispersal via convective updrafts. All *V*_term_ we measured were well below this empirical threshold (Table 2). This should facilitate the connectivity of populations in a fragmented landscape, in which convective updrafts should be more common than in closed forest. Consequently, we expected a negative correlation of *V*_term_ and the observed successful colonization of new trees between two epiphyte censuses on pasture trees over 8 years. However, there was only a signal for the bromeliad seeds due to the pronounced difference between *Catopsis* and all others, and orchid seeds showed even the reverse trend. This result probably reflects two facts: (i) interspecific differences in *V*_term_ were very small (Table 2), and (ii) successful colonization includes much more than the arrival of diaspores on a new host. There are many additional processes, some of which were investigated in this study, that mask the expected negative correlation between colonization success and *V*_term_.

Due to the structural dependence of epiphytes on trees, it is essential that seeds adhere to the bark of a potential host. We did not find a significant correlation between epiphyte abundance, or species gain, and seed adherence. In contrast, Callaway *et al.* (2002) observed that host preference of *T. usneoides* and *Polypodium polypodioides* was related to bark rugosity. Again, adherence is only one of several factors influencing successful establishment. We expected a positive correlation of bark rugosity and seed adherence and the data support this notion, although rugosity influenced adherence only over a small part of the entire spectrum. Given a certain degree of rugosity, attachment was unaffected by bark structure (Fig. 2), which suggests that relatively smooth bark is not necessarily an obstacle for adherence of bromeliad seeds. This seems to be due to different qualities of smoothness that we had not been able to measure. For example, the bark surface of teak (rugosity <2 mm) is fissured and sponge like. For the fine hairs of bromeliad comas this is apparently sufficient structure to cling to. With our test, we could only determine adherence success at first contact, but we see no reason to assume that this is not also a good proxy for the likelihood of a lasting connection. In general, trees with structured bark facilitate the attachment of bromeliad seeds and it can be expected that even small crevices also improve the likelihood of attachment in orchid seeds or fern spores as well. Only trees with very smooth bark like eucalypts, which are common plantation trees, stand out as rather problematic for seed adherence.

Even with substantial propagule dispersal in a fragmented landscape, remnant epiphyte populations may still represent ‘living dead’ (Janzen 2001) if germination and establishment were significantly impacted by less favourable microclimatic conditions in human-modified landscapes. Tree plantations are typically depicted as depauperate in epiphyte species richness (e.g. Merwin *et al.* 2003; Boelter *et al.* 2011; Carvajal-Hernández *et al.* 2014), although they may still represent a refuge in a fragmented landscape. However, being structurally less diverse than forest, plantations might provide similarly poor microclimatic conditions for germination and establishment as isolated trees due to the lack of a buffering effect of undergrowth. In a montane landscape in Ecuador, Werner and Gradstein (2008) found seedling establishment significantly reduced in isolated trees compared to forest trees. Their research focussed on tree trunks where habitat-related microclimatic differences are probably much more pronounced than in tree crowns. We conducted our germination and seedling establishment experiments in tree crowns and microclimate differed very little between tree crowns from isolated, plantation and secondary forest trees. Although tree crowns in teak plantations apparently do not offer a more forest-like microclimate the environment they provide is not unsuitable for epiphytes. The results of a study assessing the epiphyte diversity of plantations and secondary forest patches in the same study region support this statement: some teak plantations hosted quite diverse epiphyte assemblages (Einzmann and Zotz 2016). However, their value as epiphyte refuge is still debateable since timber plantations have short harvest rotation times (ca. 20 years, personal communication with plantation managers of the study region) and thus represent perpetual sinks. The expectation of higher temperatures in pasture trees compared to secondary forest trees bore out. However, the differences were not large and might be biologically irrelevant for germination. For example Marques *et al.* (2014) found optimum germination ranges of 20–30 °C in 10 out of 12 bromeliad species. Surprisingly, relative humidity was hardly lower in isolated trees than in secondary forest trees either (e.g. the mean RH in forest trees was only 2% higher than in isolated *Anacardium*) and the mean humidity in *Guazuma* even exceeded that of secondary forest trees (Table 3). Neither germination nor survival differed significantly between trees growing alone, in teak plantations or in secondary forests. The only significant signals and occasional trends we found highlighted the adaption to drier tropical climate of *T. fasciculata* and *T. flexuosa*. In contrast, a study with an epiphytic orchid in montane Costa Rica by Kartzinel et al. (2013) found cultivated trees and more open sites to be less favourable for germination. Several reports suggest that bromeliads benefit from a more open habitat (Hietz-Seifert *et al.* 1996; Dunn 2000; Barthlott *et al.* 2001; Cascante-Marín *et al.* 2006).

Clearly there is a difference in the germination and establishment success of epiphytes in modified landscapes between lowland and humid montane landscapes (Werner *et al.* 2011). Werner and Gradstein (2008) studied the establishment of vascular epiphytes on isolated trees in a tropical montane landscape. They concluded that abiotic requirements for seedlings, like microclimatic differences, will increasingly constitute a bottleneck for the persistence of epiphyte communities. Similarly, ferns of montane forests in Mexico were highly susceptible to the alteration of forest structures that lead to major microclimatic changes, whereas fern species surviving in degraded forests of the lowlands appeared to be already adapted to stressful environmental conditions (Carvajal-Hernández *et al.* 2017).

The species used in the present study were all collected in already altered landscapes, thus, the lack of a significant reduction in germination and establishment success may not really be surprising, although species differences in these ontogenetic phases were not consistent with the observed differences in species distribution along the studied rainfall gradient (Einzmann and Zotz 2017). Also, the findings regarding germination and establishment success of bromeliads cannot be representative for all bromeliads, let alone all epiphytes. In particular, more hygrophilous species will show different comportment, as extremely hygrophilous species are probably already lost in the entire study area, while moderately hygrophilous species are only found in the wetter parts (Poltz and Zotz 2011; Einzmann and Zotz 2017). The pronounced drought tolerance of *T. flexuosa* during germination and as seedling has been shown previously by Bader *et al.* (2009). The three other species in our study were less often found in the driest parts of the study region (Einzmann and Zotz 2017). Cascante-Marín *et al.* (2008) expected higher establishment of CAM bromeliads in comparison to C3 species in younger forests, but only found that all bromeliad species performed better in younger forest. However, if bromeliads fare so much better in open habitat we would have expected a clearer positive response, which was not the case. What the results do show is that the microclimatic differences in tree crowns are rather small in the different secondary habitats with no differences in germination and seedling establishment.

## Conclusions

We studied important processes of the early ontogeny of vascular epiphytes to reach a mechanistic understanding of the long-term viability of epiphyte (meta) communities in human-modified landscapes. We quantify *V*_term_ as a proxy for the capacity of long-distance dispersal of the seeds of 13 species of orchids and bromeliads and found very low values. Attachment of seeds to bark was invariant as long as grooves were at least 1 mm deep. Taken together, dispersal and connectivity may thus be a minor problem in these systems. Furthermore, microclimatic conditions in isolated trees, trees growing in groups like teak plantations or secondary forest patches were not very different. Consistent with similar microclimatic conditions between habitat types, germination and early establishment success were also very similar. Taken together, this suggests that epiphytes with anemochorous propagules can easily colonize new sites in fragmented landscapes, increasing the probability that epiphyte metacommunities are viable in the long run as long as trees are present and disturbance is limited. This is in line with findings of a number of studies that suggested that some drought-tolerant species may even do better than in closed forest.

## Sources of Funding

Funding was received from the German Academic Exchange Service (HE) and Smithsonian Tropical Research Institute (GZ).

## Contributions by the Authors

H.J.R.E. conceived and designed the experiments with input from G.Z. H.J.R.E. performed the experiments except the determinations of *V*_term_ of seeds, which were done by Maike Westhoff. H.J.R.E. analysed the data, and H.J.R.E. and G.Z. wrote the manuscript.

## Conflicts of Interest

None declared.

## Supporting Information

The following additional information is available in the online version of this article—

**Figure S1.** Example of how *V*_term_ for an individual orchid seed was estimated with a linear regression.

**Table S2.** Adherence of seeds of 10 bromeliad species on oak bark.

**Figure S3.** Sketch of the custom-built container to minimize air movement in the target area where the seeds were blown towards the trunk.

**Figure S4.** Sketch of how where microclimate measurements were conducted within the tree crowns.

**Figure S5.** Photo of a germination and an establishment plot within the crown of an *Anacardium occidentale* tree.

**Table S6.** Seed adherence and rugosity of the trunk bark of 33 species growing in rural Panama.

**Figure S7.** Boxplots of the results of the microclimate measurements.

## Supplementary Material

Supporting Information
