# Supplementary material for: Dispersal and establishment of vascular epiphytes in human-modified landscapes
Source: AoB Plants. 2017 Oct 3;9(6):plx052. doi: 10.1093/aobpla/plx052 (PMC5714248; doi:10.1093/aobpla/plx052)
Supplement: Supporting Information [file plx052_suppl_supporting_information.docx]

## Supporting Information

# Dispersal and establishment of vascular epiphytes in human-modified landscapes

Helena JR Einzmann*^,1^, Gerhard Zotz^1,2^

^1^ Department of Biology and Environmental Sciences, Carl von Ossietzky University of Oldenburg, Ammerländer Heerstraße 114–118, D-26129 Oldenburg, Germany.

^2^ Smithsonian Tropical Research Institute, Apartado Postal 0843-03092, Balboa, Ancon, Panamá, República de Panamá.

*Corresponding author. Email address: helena.einzmann@uni-oldenburg.de


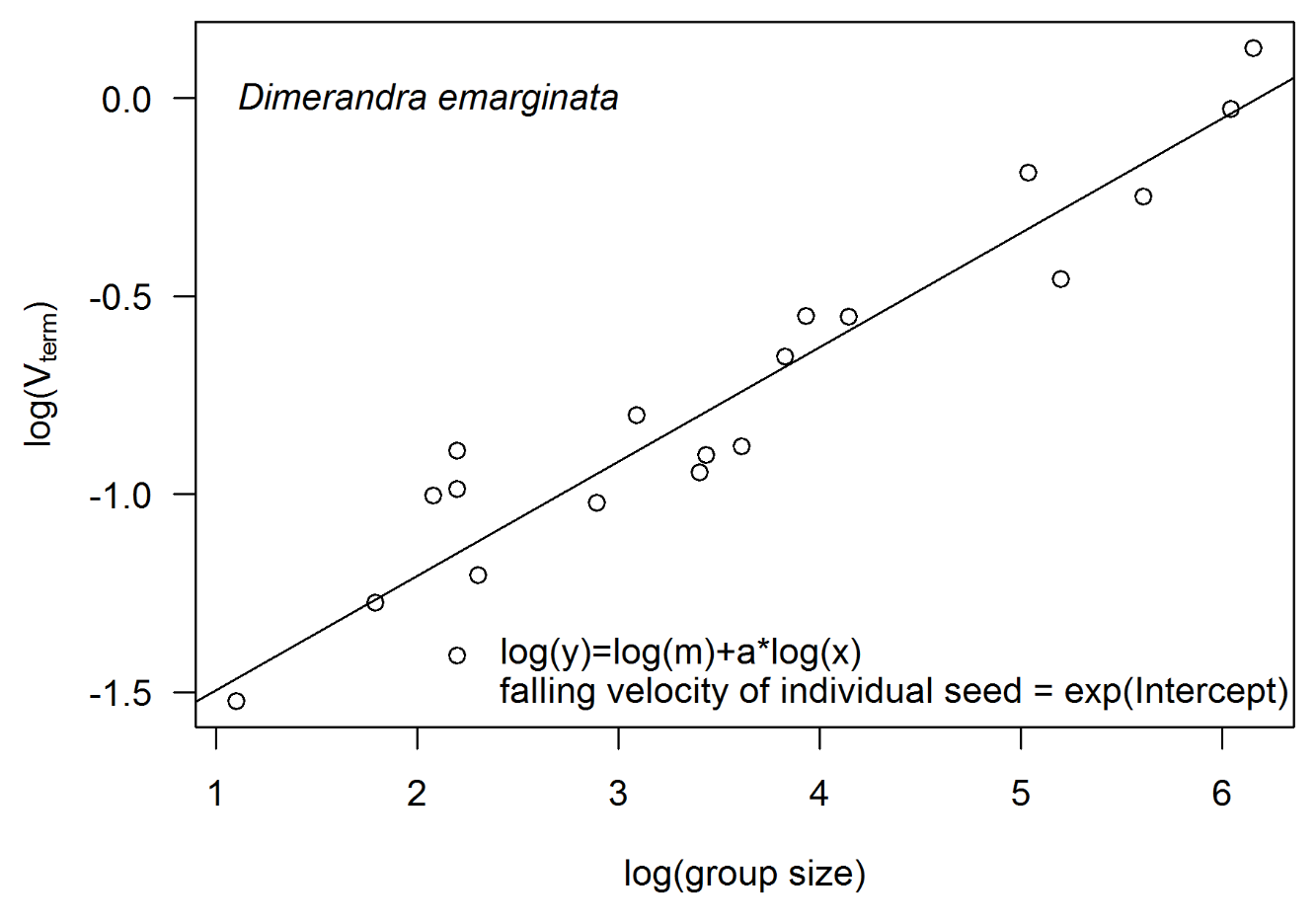


**Figure S1.** Orchid seeds fell mainly in groups. To estimate V_term_ for an individual seed of each species a linear regression of the log transformed data was applied. Here data of *Dimerandra emarginata* is shown as an example.

**Table S2.** Adherence of seeds of ten bromeliad species on oak bark (structure depth 1.1 ± 0.4 cm). Data are means ± SD, n = 3 per species.

| **Species** | **Seed adherence (%)** | | |
| --- | --- | --- | --- |
| *Catopsis nitida* (Hook.) Griseb. | 100 | ± | 0 |
| *Catopsis nutans* (Sw.) Griseb. | 97 | ± | 5 |
| *Guzmania monostachia* (L.) Rusby ex Mez | 100 | ± | 0 |
| *Tillandsia balbisiana* Schult. & Schult.f. | 93 | ± | 5 |
| *Tillandsia brachycaulos* Schltdl. | 97 | ± | 5 |
| *Tillandsia elongata* Kunth | 100 | ± | 0 |
| *Tillandsia fasciculata* Sw. | 97 | ± | 5 |
| *Tillandsia spiculosa* Griseb. | 93 | ± | 5 |
| *Tillandsia subulifera* Mez | 100 | ± | 0 |
| *Vriesea ringens* (Griseb.) Harms | 100 | ± | 0 |


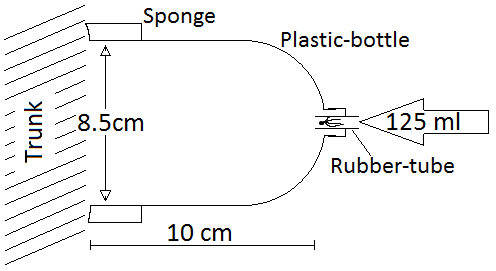


**Figure S3.** Sketch of the custom-built container to minimise air movement in the target area where the seeds were blown towards the trunk.


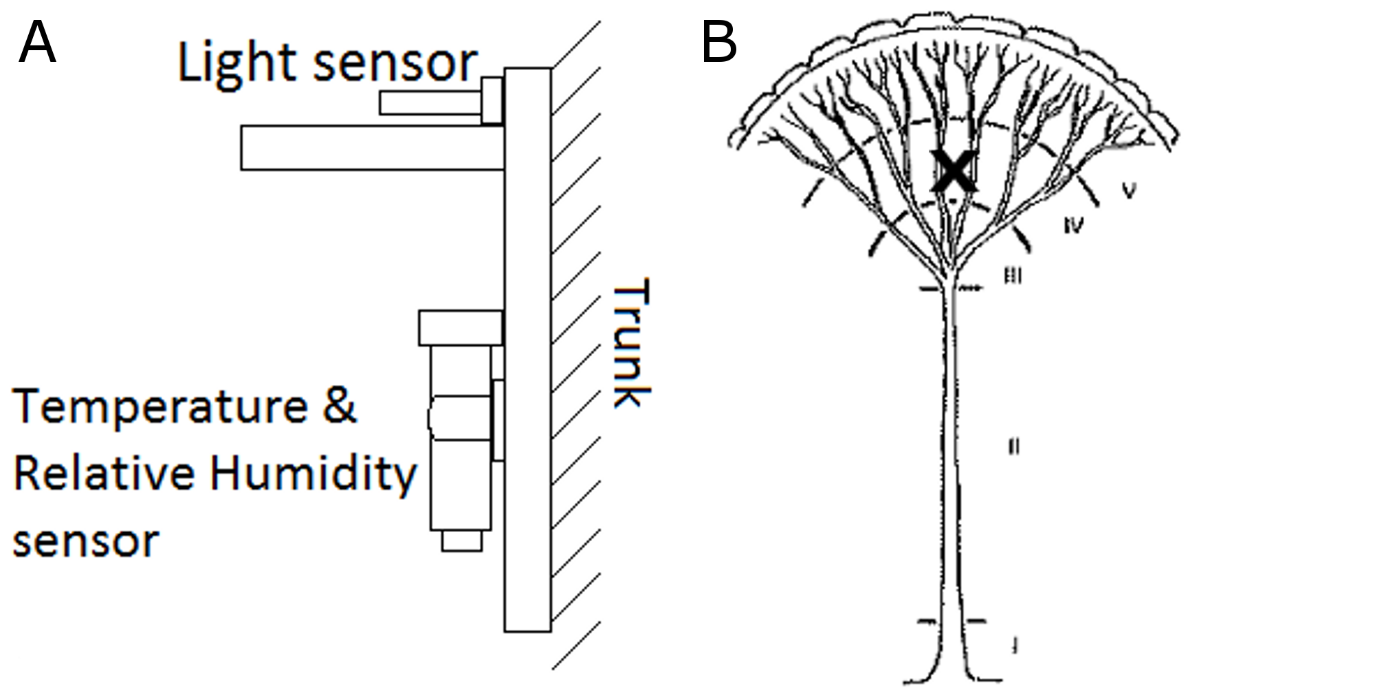


**Figure S4.** Microclimate measurements were conducted using (A) two measuring devices mounted on a wooden construction that was fixed (B) in the centre of the tree crown, which would correspond to a position in Johansson Zone IV (modified after Johansson 1974).

**
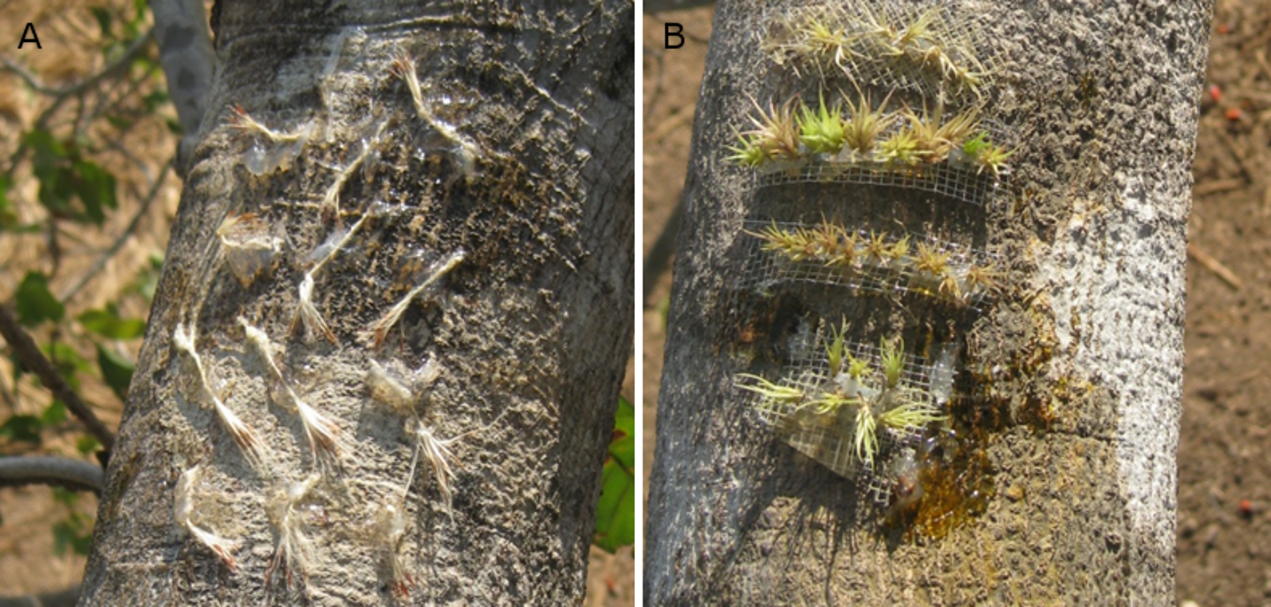
**

**Figure S5.** (A) Germination and (B) establishment plots on *Anacardium occidentale*. The species in the germination plot are from top to bottom: *Tillandsia fasciculata*, *T. elongata*, *T. flexuosa* and *T. balbisiana*. In the establishment plot there are from top to bottom: *T. balbisiana*, *T. flexuosa*, *T. fasciculata*, *T. elongata.*

**Table S6.** Seed adherence and rugosity of the trunk bark of 33 species growing in rural Panama. Data are means ± SD, n = 5 per species.

| Taxon | Seed adherence  (%) | | | Bark structure  (depth in mm) | | |
| --- | --- | --- | --- | --- | --- | --- |
| Anacardiaceae |  |  |  |  |  |  |
| *Anacardium excelsum* (Bertero ex Kunth) Skeels | 62 | ± | 8 | 4.8 | ± | 1.3 |
| *Anacardium occidentale* L. | 66 | ± | 13 | 1.0 | ± | 0.7 |
| *Mangifera indica* L. | 77 | ± | 16 | 6.7 | ± | 1.9 |
| Arecaceae |  |  |  |  |  |  |
| *Cocos nucifera* L. | 66 | ± | 16 | 2.7 | ± | 1.6 |
| Bignoniaceae |  |  |  |  |  |  |
| *Crescentia cujete* L. | 82 | ± | 14 | 1.9 | ± | 1.5 |
| *Tabebuia rosea* (Bertol.) Bertero ex A.DC. | 64 | ± | 28 | 4.0 | ± | 1.0 |
| Boraginaceae |  |  |  |  |  |  |
| *Cordia alliodora* (Ruiz & Pav.) Oken | 82 | ± | 14 | 6.8 | ± | 1.6 |
| Burseraceae |  |  |  |  |  |  |
| *Bursera simaruba* (L.) Sarg. | 2 | ± | 2 | 0.1 | ± | 0.1 |
| Chrysobalanaceae |  |  |  |  |  |  |
| *Licania arborea* Seem. | 74 | ± | 16 | 1.5 | ± | 1.3 |
| Combretaceae |  |  |  |  |  |  |
| *Terminalia catappa* L. | 84 | ± | 13 | 2.6 | ± | 1.1 |
| Dilleniaceae |  |  |  |  |  |  |
| *Curatella americana* L. | 74 | ± | 11 | 4.0 | ± | 1.2 |
| Lamiaceae |  |  |  |  |  |  |
| *Gmelina arborea* Roxb. | 36 | ± | 15 | 0.5 | ± | 0.6 |
| ** Tectona grandis* L.f. | 78 | ± | 19 | 0.9 | ± | 0.7 |
| Lauraceae |  |  |  |  |  |  |
| *Ocotea veraguensis* (Meisn.) Mez | 7 | ± | 12 | 2.5 | ± | 1.0 |
| Leguminosae |  |  |  |  |  |  |
| *Andira inermis* (Wright) DC. | 76 | ± | 15 | 4.4 | ± | 0.8 |
| *Cassia moscata* Kunth | 76 | ± | 15 | 3.7 | ± | 1.4 |
| *Diphysa americana* (Mill.) M.Sousa | 78 | ± | 16 | 5.7 | ± | 3.0 |
| *Enterolobium cyclocarpum* (Jacq.) Griseb. | 82 | ± | 8 | 1.5 | ± | 0.8 |
| *Erythrina berteroana* Urb. | 60 | ± | 23 | 3.6 | ± | 0.8 |
| *Gliricidia sepium* (Jacq.) Walp. | 74 | ± | 11 | 1.7 | ± | 0.4 |
| *Hymenaea courbaril* L. | 68 | ± | 16 | 1.4 | ± | 1.7 |
| Malpighiaceae |  |  |  |  |  |  |
| *Byrsonima crassifolia* (L.) Kunth | 81 | ± | 13 | 2.3 | ± | 1.3 |
| Malvaceae |  |  |  |  |  |  |
| *Guazuma ulmifolia* Lam. | 86 | ± | 20 | 2.3 | ± | 0.7 |
| *Luehea seemannii* Triana & Planch | 66 | ± | 23 | 1.7 | ± | 1.3 |
| *Sterculia apetala* (Jacq.) H.Karst. | 68 | ± | 27 | 0.7 | ± | 0.7 |
| Melastomataceae |  |  |  |  |  |  |
| *Miconia argentea* (Sw.) DC. | 72 | ± | 22 | 3.2 | ± | 1.3 |
| Meliaceae |  |  |  |  |  |  |
| *Cedrela fissilis* Vell. | 76 | ± | 25 | 4.7 | ± | 3.5 |
| Moraceae |  |  |  |  |  |  |
| *Ficus* sp | 70 | ± | 21 | 0.1 | ± | 1.3 |
| Myrtaceae |  |  |  |  |  |  |
| ** Eucalyptus* sp | 18 | ± | 17 | 0.1 | ± | 1.3 |
| Pinaceae |  |  |  |  |  |  |
| ** Pinus caribaea* Morelet | 70 | ± | 18 | 8.8 | ± | 4.2 |
| Rubiaceae |  |  |  |  |  |  |
| *Genipa americana* L. | 28 | ± | 27 | 0.5 | ± | 0 |
| Urticaceae |  |  |  |  |  |  |
| *Cecropia* sp | 40 | ± | 41 | 0.1 | ± | 0.1 |
| Undetermined |  |  |  |  |  |  |
| Spec1 | 68 | ± | 22 | 3.6 | ± | 0.4 |

* Species commonly planted as monoculture in the study region


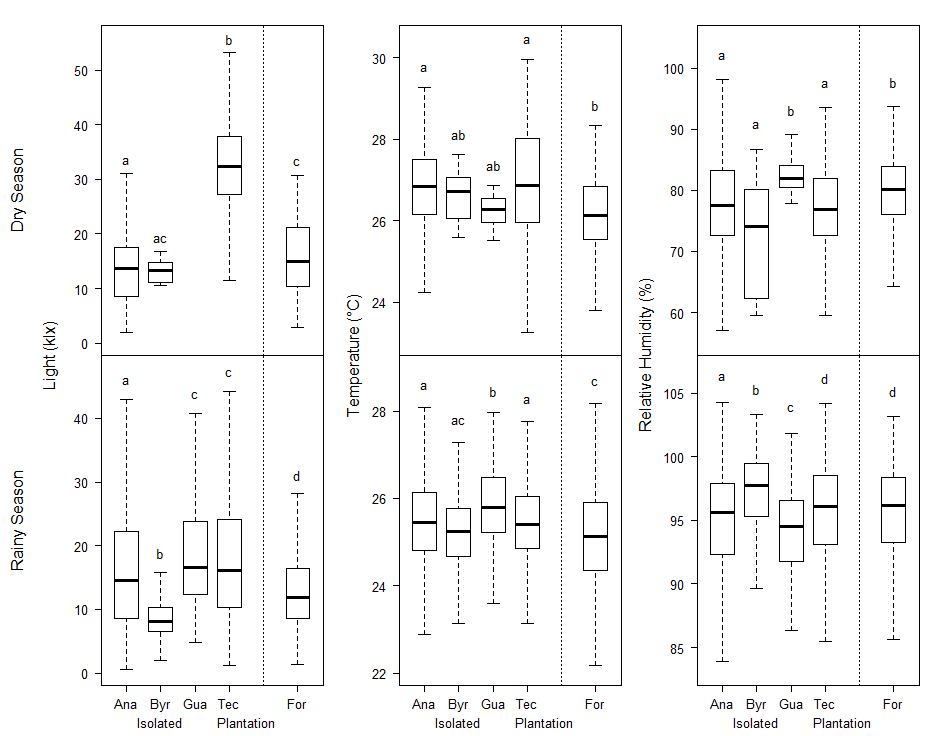


**Figure S7.** Light, temperature, and relative humidity within tree crowns in three different habitat types. Measurements were conducted in isolated trees (*Anacardium occidentale* – n = 9 for light, temperature and relative humidity in both seasons, *Byrsonima crassifolia* – n = 1 for all cases, *Guazuma ulmifolia* – n = 1 for all cases), teak plantations (*Tectona grandis* – n = 3 / 5 for all variables in dry seasons / rainy season), and secondary forest patches (various undetermined species within the forest patches – n = 5 / 6 for all variables in dry season / rainy season). Data from months of transition from dry to rainy season and vice versa were excluded from analyses (KW: *P* < 0.001, NT: *P* < 0.05). The box plots depict the median as bold black bar. The box represents the inner quartile range (IQR), while whiskers extend to extreme values within the first quartile -1.5 × IQR and, respectively, within the third quartile +1.5 × IQR. Outliers are not displayed.
